# *BRCC3* ‐Associated Syndromic Moyamoya Angiopathy Diagnosed Through Clinical RNA Sequencing

**DOI:** 10.1111/cge.14650

**Published:** 2024-11-17

**Authors:** Myrrhe Venema, Fatimah Albuainain, Rachel Schot, Bob Roozenbeek, Frank Sleutels, Tjakko van Ham, Tahsin Stefan Barakat

**Affiliations:** ^1^ Department of Clinical Genetics Erasmus MC University Medical Center Rotterdam The Netherlands; ^2^ Department of Neurology Erasmus MC University Medical Center Rotterdam The Netherlands

**Keywords:** *BRCC3*, missing heritability, moyamoya angiopathy, neurodevelopmental disorders, RNA‐seq, Xq28 deletion

## Abstract

Moyamoya angiopathy is a cerebral vasculopathy causing progressive stenosis of the internal carotid arteries and the compensatory development of collateral blood vessels, leading to brain ischemia and an increased risk of cerebral haemorrhage. Although multiple non‐genetic causes have been associated with moyamoya syndrome, it can also be associated with rare genetic syndromes. Moyamoya Disease 4, characterised by a short stature, hypergonadotropic hypogonadism and facial dysmorphism (MYMY4, OMIM #300845), also referred to as *BRCC3‐*associated moyamoya syndrome, has so far been described in 11 individuals. Here, we describe a 23‐year‐old male presenting with moyamoya syndrome, global developmental delay and intellectual disability, epilepsy, short stature and dysmorphic features, who after > 17 years of uninformative diagnostics was diagnosed with *BRCC3‐*associated moyamoya syndrome after clinical RNA‐seq. Transcriptome analysis showed reduced expression of the likely disease‐causing gene *BRCC3* in patient‐derived fibroblasts, which was subsequently found to be caused by a ~ 26 kb Xq28 deletion. We furthermore review all reported cases of *BRCC3‐*associated moyamoya syndrome, further delineating this clinical entity.

## Graphical abstract

We describe a new individual diagnosed with *BRCC3*‐associated syndromic moyamoya angiopathy through clinical RNA sequencing. We provide further clinical details on the phenotypic spectrum of this rare disease while highlighting the role of RNA‐seq as a complementary diagnostic tool.

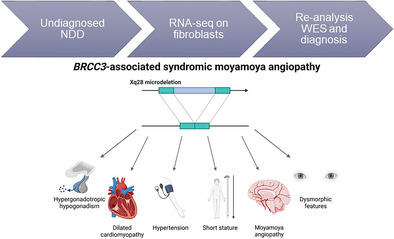

## 1 Introduction

Moyamoya syndrome is an angiopathy of the cerebral vasculature, characterised by progressive stenosis of the intracranial internal carotid arteries, leading to reduced blood flow in the anterior circulation of the brain and compensatory development of new blood vessels [1]. The formation of collateral vessels creates a typical pattern on angiography, resembling ‘a puff of cigarette smoke’, or *moyamoya* in Japanese [1]. Symptoms include those caused by brain ischemia due to stenosis, including transient ischemic attacks, ischemic strokes and cerebral haemorrhage [1].

While moyamoya is associated with various risk factors, there are associated genetic disorders [1]. One of these is caused by an Xq28 microdeletion containing the *BRCC3* gene and the flanking bicistronic locus encoding transcripts for the *CMC4* and *MTCP1* genes. This specific moyamoya syndrome (MYMY4, OMIM #300845) is characterised by a short stature, hypergonadotropic hypogonadism and facial dysmorphism, amongst other symptoms, including dilated cardiomyopathy, hypertension and early‐onset cataracts [2, 3, 4]. So far, 11 individuals have been reported, providing challenges for clinical management, as the phenotypical spectrum has not yet been fully defined. Here, we describe an individual with a *BRCC3‐*associated moyamoya syndrome, which, after a longstanding diagnostic odyssey, was diagnosed by clinical RNA sequencing (RNA‐seq).

## 2 Methods

### 2.1 Recruitment and Genomic Investigations

The individual was clinically investigated, and genetic analyses were performed in a clinical setting. Informed consent was obtained for all diagnostics, including written informed consent from proband and parents for publication of medical data and photographs, in line with the Declaration of Helsinki. Genetic analysis and clinical RNA‐seq on fibroblasts were performed as described [5, 6]. Use of genome‐wide technologies for diagnostic purposes was previously approved (Institutional‐review‐board MEC‐2012‐387). For PCR validation, a deletion‐specific PCR was performed using routine procedures and primers CMC4DEL_F: ACGATTCAAGTTGGCGGACTA; CMC4DELNOR_R: GTGTGACCTCTTAGAAAATTGGGC; and CMC4DELMUT_R: ATTCAGGTACGTTAAGTGTGTGT. The Graphical Abstract figure was created in BioRender. Venema, M. (2024) https://BioRender.com/h80q580.

### 2.2 Clinical Description

**FIGURE 1:**
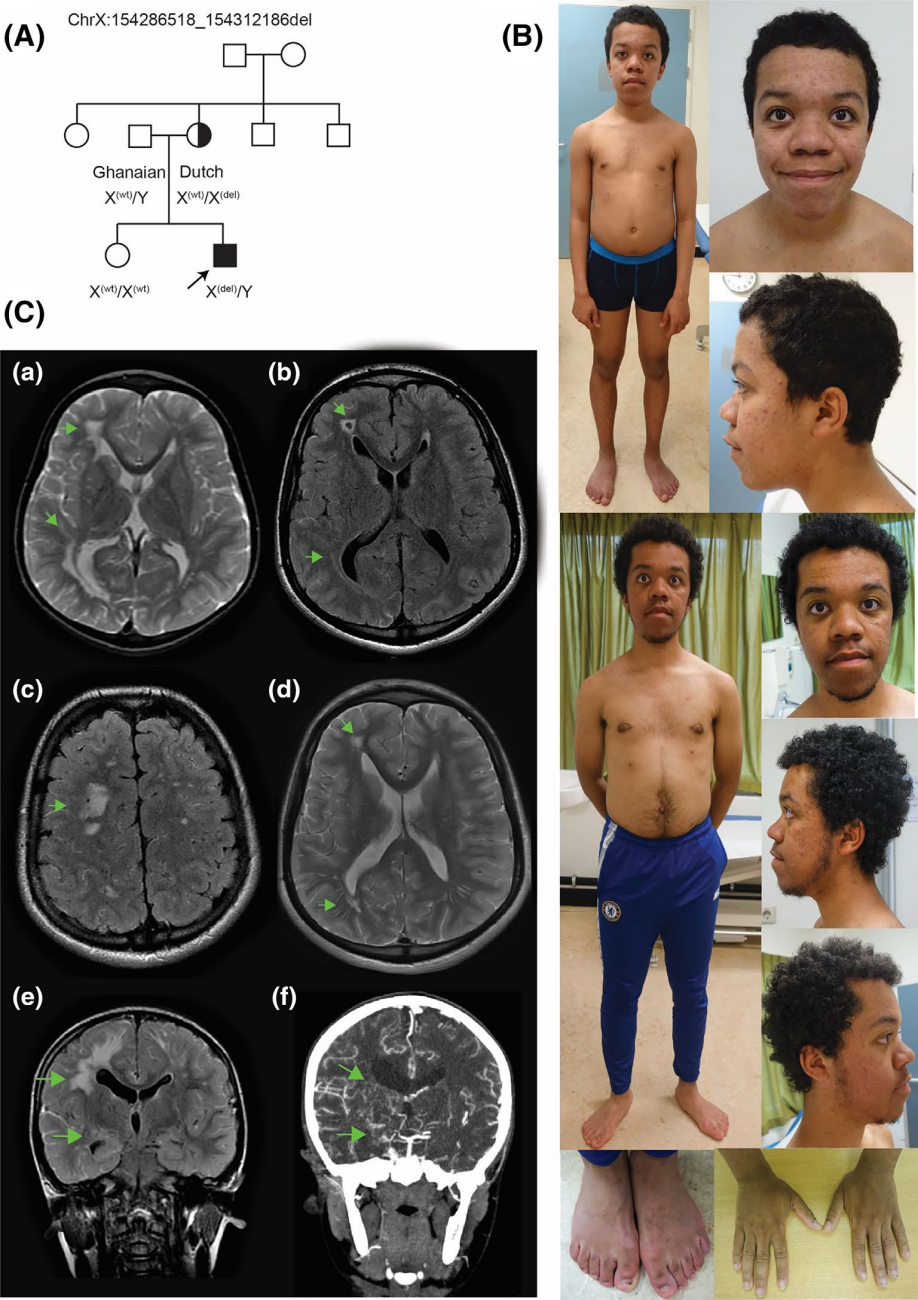
Clinical overview. (A) Family pedigree. (B) Photographs at 16 years (top) and 23 years (bottom). (C) T2‐weighted (a, d) and T2‐FLAIR (b, c, e) brain‐MRI at 6 years (a, e) and 17 years (b–d) in the axial (a–d) or coronal plane (e) showing generalised bilateral white matter abnormalities at supratentorial, right‐sided periventricular and subcortical regions and the left‐sided frontal area (arrows). Liquor spaces are asymmetrically enlarged without progression. Cerebral CT angiography at 8 years (f), showing bilateral narrowing of the internal carotid arteries, most pronounced on the right and at the *T*‐junction. There is a distinct puff of smoke appearance, with prominent lenticulostriate arteries (arrows).

The affected individual is a 23‐year‐old male, who presented with a history of global developmental delay and mild intellectual disability. He was the second child, born to non‐consanguineous parents (Figure 1A). Family history was unremarkable. Pregnancy and delivery were uncomplicated. Birth weight and length were 4 kg (+1.3SD) and 50 cm (+0.1SD), respectively. Postnatally, an enlarged fontanel triggered a cerebral ultrasound, showing a benign communicating hydrocephalus. Motor development was normal, with ambulation at 1 year. Speech development was delayed, with no babbling at 18 months. While a bilateral conductive hearing loss of 30 dB was found, no speech improvement occurred after tonsillectomy and tympanostomy tube placement, although speech eventually developed at 9 years, with the verbal abilities of a 6‐year‐old.

At 6 years, examination showed a height of 118 cm (−0.4SD), a weight of 22 kg (+0.4SD) and a head circumference of 54.8 cm (+2SD) as well as dysmorphic features including hypertelorism and bilateral supernumerary nipples (Figure 1B). Except for developmental delay, no focal neurological symptoms were present. Brain‐MRI showed periventricular and subcortical white matter lesions, mild ventricle dilatation and diffuse white matter loss, hypothesised as likely caused by prenatal infections.

At 7 years, seizure‐like symptoms developed, with absences, muscle jerkings and restless sleep with lip‐smacking. EEG showed generalised epileptiform activity, with maximal epileptic activity alternating between the left parietal and central regions. At 8 years, brain‐MRI showed similar white matter lesions, dilated ventricles and diffuse white matter loss, with left frontal lobe cortical loss and periventricular gliosis of the occipital lobe. Furthermore, CTA of the brain showed bilateral stenosis of the internal carotid arteries, interpreted as signs of cerebral vasculitis or moyamoya angiopathy (Figure 1C). This finding was then concluded as a more likely cause of the MRI abnormalities rather than prenatal incidents. Retrospective MRI analysis indicated that internal carotid artery stenosis potentially had already started earlier. Cardiology follow‐up showed no signs of vasculitis but showed hypertension. At 9 years, neuropsychological examination established below‐average cognitive and verbal abilities, with a TIQ of 61. Furthermore, signs of a frontal syndrome, with reduced initiative, highly associative behaviour and perseverations were found, with regression of non‐verbal abilities.

**FIGURE 2:**
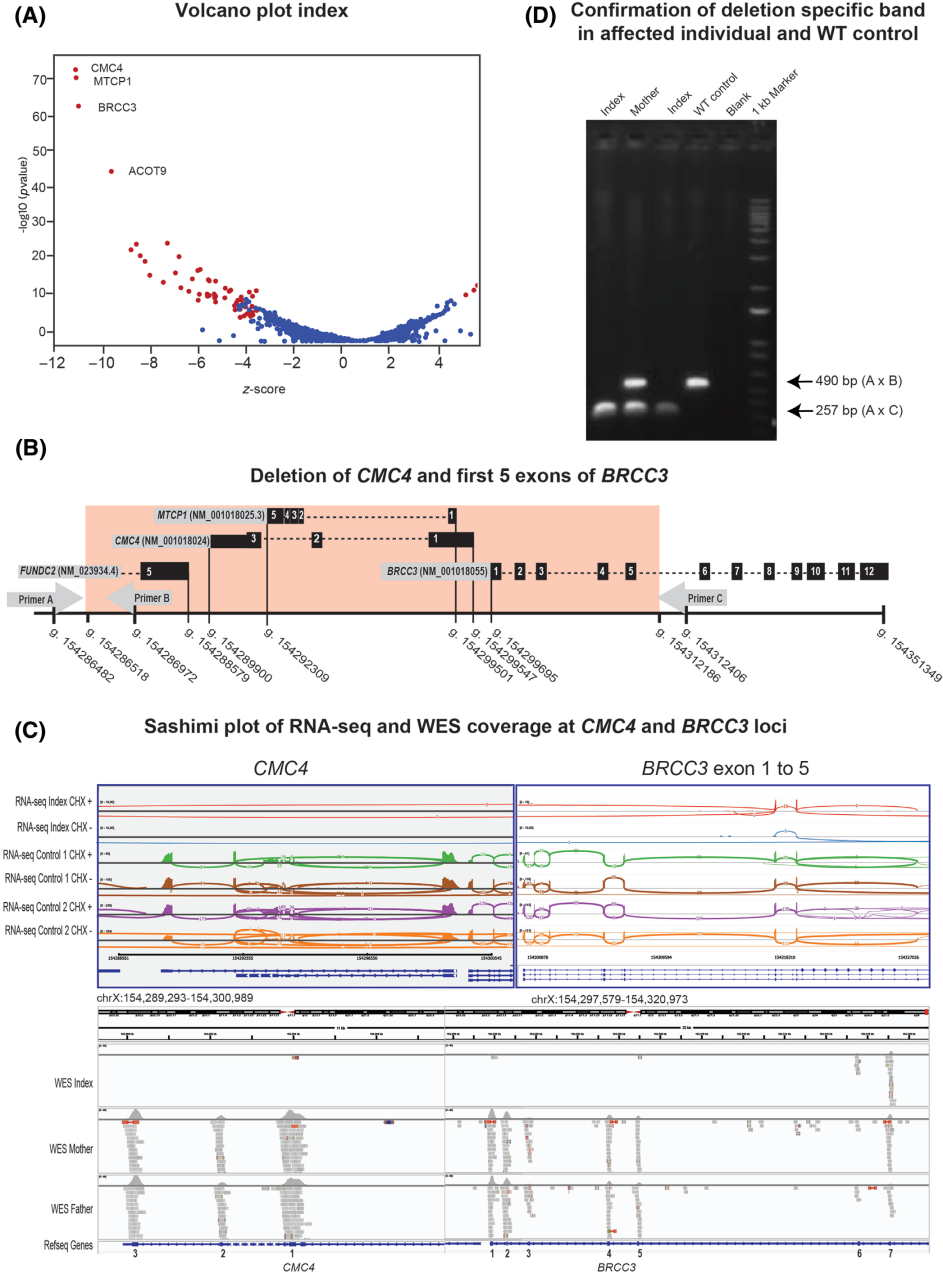
RNA‐seq identifying a disease‐causing Xq28 deletion. (A) Volcano plot showing *z*‐scores at the gene level and log10 (*p* values) for all assessed genes by RNA‐seq in the index. Here, *CMC4, MTCP1* and *BRCC3* showed the most striking downregulation (*z*‐scores < −10); other genes did not show significant downregulation (*z*‐score < −4), except for *ACOT9* (*z*‐score − 8). *ACOT9* downregulation is likely caused by a maternally inherited hemizygous frameshift variant. (B) Scheme of the ~25.6 kb Xq28 deletion, showing *CMC4, MTCP1, BRCC3* and *FUNDC2*, with coordinates, deletions and primers indicated. (C) IGV‐browser showing Sashimi plots at *CMC4* and *BRCC3* from the index and controls, for RNA‐seq of fibroblasts cultured with or without cyclohexamide (CHX). Also shown are WES data for the same region for index and unaffected parents, and schematic showing the expressed transcripts of *CMC4* and *BRCC3*. The index has no DNA and RNA‐seq reads mapping to *CMC4* and exons 1–5 of *BRCC3*, indicating the presence of a maternally inherited hemizygous deletion encompassing *CMC4, MTCP1* and *BRCC3* (exons 1–5). (D) PCR validation of deletion. Shown are PCR products for the index (2×), mother, unrelated and negative control (blank). PCR was performed using primer A and a mixture of primers B and C (see panel B), giving rise to a 491 bp wild‐type band and a 257 bp deletion‐specific band. PCR validation confirmed the maternally inherited hg19: ChrX: 154286518_154312186del in the index.

At 16 years, the height was 156 cm (−3SD) and the head circumference was 57.2 cm (+0.3SD). Dysmorphic features included hypertelorism, downslanting palpebral fissures, mild proptosis, an elongated face with a prominent chin, small ears, pectus excavatum, bilateral supernumerary nipples, small hands and feet, cubitus valgus and acne‐like lesions (Figure 1B). Extended genetic investigations then, and re‐analysis 5 years later, including SNP array, metabolic investigations, RAS‐opathy gene panel and trio whole exome sequencing (WES) focussing on intellectual disability and multiple congenital anomaly genes, complete exome analysis and analysis for mosaicism in fibroblasts did not identify a disease‐explaining variant (Table S1). Finally, RNA‐seq on fibroblast‐derived RNA revealed the significant downregulation of *CMC4*, *MTCP1*, *BRCC3* and *ACOT9* (Figure 2A), the latter being explained by a maternally inherited frameshift variant in *ACOT9*, a gene without the OMIM phenotype. Retrospective WES‐data analysis revealed a maternally inherited, hemizygous Xq28 deletion of ~25.6 kb (hg19: ChrX:154286518_154312186del) (Figure 2C), which was PCR‐validated (Figure 2D). This deletion contained *CMC4* and *MTCP1*, the first five exons of *BRCC3* and part of the 3' UTR of *FUNDC2* and was absent in the unaffected sister. Further family segregation was not performed. Maternal X‐inactivation analysis showed no significant skewing (56% and 63% methylated in the two experiments; not shown).

## 3 Discussion

Here, we report an individual with *BRCC3*‐associated moyamoya syndrome, which, after a longstanding, non‐informative diagnostic journey, was finally diagnosed using clinical RNA‐seq. Previous evidence pointed to *BRCC3* as the most likely causative gene in this disorder. By comparing genetic variants amongst nine patients, a critical region of 3362 bp (*MTCP1* exon 1 and *BRCC3* exons 1–3) was identified [3]. BRCC3 (BRCA1/BRCA2 containing complex, subunit 3) is an E3 ubiquitin ligase involved in various cellular processes, including DNA damage repair, cell‐cycle regulation and inflammatory responses [7, 8, 9]. Inhibiting miRNAs against BRCC3 have been found to be upregulated in non‐syndromic moyamoya [10]. Additionally, inhibition of *BRCC3* in zebrafish resulted in defective angiogenesis, suggesting a pathophysiological role of BRCC3 in moyamoya angiopathy. In contrast, *MTCP1* knockouts did not affect angiogenesis [3].

Including ours, 12 individuals with *BRCC3*‐associated moyamoya syndrome have been described. Some symptoms are prevalent, including moyamoya angiopathy (10/12), short stature (12/12) and hypertension (6/12). Contrarily, developmental delay/intellectual disability (4/12) and epilepsy (2/12) are less common, but were present in our patient (Table 1) [2, 3, 4, 11]. This might be explained by white matter lesions and gliosis already present at young age, signifying that symptomatic damage could have occurred early on due to moyamoya angiopathy. Alternatively, the underlying genetic defect might directly impact cognition. Notably, our patient did not show signs of hypergonadotropic hypogonadism (7/12). Furthermore, cardiological assessment did not show dilated cardiomyopathy, found in (4/12). Though all individuals in Table 1 have smaller Xq28 deletions containing *BRCC3* and *CMC4/MTCP1*, larger Xq28 deletions are also reported. These may include the gene *F8*, resulting in the severe haemophilia A and moyamoya (SHAM) syndrome [12]. Of the nine individuals described with SHAM, seven had confirmed Xq28 deletions including *F8, BRCC3* and *MTCP1* [12, 13, 14, 15, 16, 17, 18]. Haemophilia A (7/7), moyamoya angiopathy (5/7), stroke (6/7), hypertension (3/7), hypergonadotropic hypogonadism (2/7), learning disability/developmental delay (2/7), facial dysmorphism (3/7), short stature (3/7) and premature grey hair (1/7) were their main features [12, 13, 14, 15, 16, 17, 18]. Additionally, two female symptomatic carriers were described with haemophilia, hypertension and cardiac abnormalities, though also healthy carriers have been described [18, 19]. Hence, it becomes evident that there is phenotypic overlap between SHAM and MYMY4.

**TABLE 1:** Clinical characteristics.

Clinical phenotype	HPO number	Affected individual (this paper), *n* = 1	Affected individuals (Hervé et al.), *n* = 5	Affected individuals (Miskinyte et al.), *n* = 4	Affected individual (Pyra et al.), *n* = 1	Affected individual (Rodriguez‐Gil), *n* = 1^a^	Affected individuals (total), *n* = 12
Neurological symptoms and manifestations							
Developmental delay/intellectual disability	0012758/0001249	1/1	0/5	2/4	NA	1/1	4/12
Moyamoya angiopathy	0011834	1/1	4/5	4/4	1/1	0/1	10/12
Stroke	0001297	0/1	4/5	3/4	1/1	NA	8/12
Seizures/epilepsy	0001250	1/1	0/5	1/4	0/1	NA	2/12
Endocrinological manifestations							
Hypergonadotropic hypogonadism	0000815	NA	5/5	2/4	0/1	0/1	7/12
(Partial) GH deficiency	0000824	NA	2/5	2/4	1/1	0/1	5/12
Cardiovascular manifestations							
Dilated cardiomyopathy	0001644	0/1	3/5	0/4	1/1	0/1	4/12
Hypertension	0000822	1/1	0/5	3/4	1/1	1/1	6/12
Dysmorphic features							
Short stature	0004322	1/1	5/5	4/4	1/1	1/1	12/12
Hypertelorism	0000316	1/1	5/5	2/4	NA	1/1	9/12
Long philtrum	0000343	0/1	5/5	2/4	NA	1/1	8/12
Mild ptosis	0000508	0/1	5/5	2/4	NA	1/1	8/12
Small hands/feet	0200055/0001773	1/1	5/5	NA	NA	1/1	7/12
Premature greying	0002216	0/1	5/5	1/4	NA	0/1	6/12
Ocular manifestations							
Early‐onset cataract	0000518	0/1	4/5	0/4	0/1	0/1	4/12

^a^ Individual also had an insertion of 61.4 kb duplicated 7p22.3 chromosomal material on the X‐chromosome.

Our individual is the first diagnosed with *BRCC3*‐associated moyamoya syndrome through clinical RNA‐seq, where the deletion was identified after RNA‐seq showed downregulation of *BRCC3*, *CMC4* and *MTCP1*. Retrospective WES analysis confirmed the Xq28 deletion, which had previously gone unnoticed due to regional sequencing noise that prevented its detection in the diagnostic pipeline. Small copy number variations (CNVs) often prove challenging for routine diagnostic tools, as SNP‐array resolution depends on probe spacing, and the accuracy of WES CNV detection may vary depending on CNV size and analysis software [20]. RNA‐seq may offer complementary diagnostic options in such instances. The main advantage is that next to sequence information, RNA‐seq assesses gene expression [5]. Thus, CNVs leading to aberrant expression profiles can be detected using RNA‐seq, as exemplified here. Several studies have already reported an increased diagnostic yield when combining RNA‐seq with WES in neuromuscular or NDD cohorts [21, 22]. We reported that clinical RNA‐seq improved the diagnostic yield of undiagnosed NDDs by 13% (9/67). Two of these cases also concerned deletions that were previously missed and confirmed through retrospective WES analysis, similar to this case [5].

In conclusion, we report an individual with BRCC3‐associated moyamoya syndrome (MYMY4, OMIM #300845) and provide further clinical data to expand the phenotypical characteristics of this syndrome. Additionally, we highlight the role of RNA‐seq as a complementary diagnostic modality.

## Author Contributions

M.V., F.A., B.R. and T.S.B. performed clinical phenotyping. R.S., F.S. and T.v.H. performed genomic investigations. M.V., F.A. and T.S.B. wrote the manuscript, with input from all authors. T.S.B. conceived and supervised the study.

## Conflicts of Interest

The authors declare no conflicts of interest.

### Peer Review

The peer review history for this article is available at https://www.webofscience.com/api/gateway/wos/peer‐review/10.1111/cge.14650.

## Supporting information


**Table S1.** Overview of genetic variations found in the affected individual.

## Data Availability

All clinical data are presented herein. All data generated or analysed during this study are included in this published article, except raw sequencing data that due to privacy regulations and given consent, cannot be publically made available.
