# Supplementary material for: BRCC3 ‐Associated Syndromic Moyamoya Angiopathy Diagnosed Through Clinical RNA Sequencing
Source: Clin Genet. 2024 Nov 17;107(3):341–7. doi: 10.1111/cge.14650 (PMC11790519; doi:10.1111/cge.14650)
Supplement: Supplementary file 1 — Table S1. Overview of genetic variations found in the affected individual. [file CGE-107-341-s001.docx]

**Supplementary Table 1: Overview of genetic variations found in the affected individual**

| Genetic variant | Classification | OMIM phenotype |
| --- | --- | --- |
| Duplication of 343 kb on chromosome region 5q13.4, includes *COX7C* (paternally inherited)  arr 5q14.3(85,635,230-85,978,500)x3 (hg18) | VUS | No phenotype |
| -NM_001099857.2(*IKBKG*):c.169G>A, p.(Glu57Lys) (hemizygous, maternally inherited) | VUS | OMIM #300636 (Immunodeficiency 33); OMIM #300291 (Ectodermal dysplasia and immunodeficiency 1) |
| -NM_001164586.1(*IGFN1*):c.2704G>C, p.(Gly902Arg); (maternally inherited)  -NM_001164586.1(*IGFN1*):c.8609delG, p.(Gly2870fs) (paternally inherited) | VUS | No phenotype |
| NM_001330259.1(*ACOT9*):c.557delC, p.(Thr186fs4) (hemizygous, maternally inherited) | VUS | No phenotype |
| -NM_004961.3(*GABRE*):c.664G>A, p.(Glu222Lys), (hemizygote, maternally inherited)  Population frequency: 0.0044088% (gnomAD v2.1; ALL, of which 3x hemizygous) | VUS | No phenotype |
| Deletion of 25 kb on chromosome region Xq28, includes *BRCC3, CMC4* and *MTCP1* (maternally inherited) hg19: ChrX: 154286518_154312186del | Likely pathogenic | OMIM #300845 (Moyamoya disease 4) |
